# Transcriptome analysis of the genes regulating phytohormone and cellular patterning in *Lagerstroemia* plant architecture

**DOI:** 10.1038/s41598-018-33506-8

**Published:** 2018-10-11

**Authors:** Yiqian Ju, Lu Feng, Jiyang Wu, Yuanjun Ye, Tangchun Zheng, Ming Cai, Tangren Cheng, Jia Wang, Qixiang Zhang, Huitang Pan

**Affiliations:** Beijing Key Laboratory of Ornamental Plants Germplasm Innovation & Molecular Breeding, National Engineering Research Center for Floriculture, Beijing Laboratory of Urban and Rural Ecological Environment, Key Laboratory of Genetics and Breeding in Forest Trees and Ornamental Plants of Ministry of Education, School of Landscape Architecture, Beijing Forestry University, Beijing, 100083 China

## Abstract

Plant architecture is a popular research topic because plants with different growth habits that may generate economic or ornamental value are in great demand by orchards and nurseries. However, the molecular basis of the architecture of woody perennial plants is poorly understood due to the complexity of the phenotypic and regulatory relationships. Here, transcriptional profiling of dwarf and non-dwarf crapemyrtles was performed, and potential target genes were identified based on the phenotype, histology and phytohormone metabolite levels. An integrated analysis demonstrated that the internode length was explained mainly by cell number and secondarily by cell length and revealed important hormones in regulatory pathway of *Lagerstroemia* architecture. Differentially expressed genes (DEGs) involved in phytohormone pathways and cellular patterning regulation were analysed, and the regulatory relationships between these parameters were evaluated at the transcriptional level. Exogenous indole-3-acetic acid (IAA) and gibberellin A_4_ (GA_4_) treatments further indicated the pivotal role of auxin in cell division within the shoot apical meristem (SAM) and suggested an interaction between auxin and GA_4_ in regulating the internode length of *Lagerstroemia*. These results provide insights for further functional genomic studies on the regulatory mechanisms underlying *Lagerstroemia* plant architecture and may improve the efficiency of woody plant molecular breeding.

## Introduction

The term ‘architecture’ in plants refers to the spatial arrangement of individual organs^1^ and is one of the most important characters in crop, horticultural and ornamental plants. Developing engineering or breeding programmes designed to produce an ideal plant architecture with desirable traits that generate economic benefits has become a crucial integrated goal of plant breeding^2^. The regulatory mechanisms underlying plant architecture have long been investigated in herbaceous model systems, including *Arabidopsis thaliana*, pea and rice^3–5^. However, the mechanisms in woody perennial plants remain poorly understood, although the establishment of different growth habits in trees is in great demand by orchards and nurseries^6,7^.

Previous studies have shown that branch length is a critical determinant of plant architecture^8^. The other major determinant of plant architecture is shoot branching^9^. Further investigations have shown that branch length is determined by the interplay between two cellular components of growth: cell size and cell number^10–12^. Sachs (1965) reported the essential role of sub-apical meristematic activity in the growth of shoots^13^, and numerous studies have focused on meristems to explore their roles in shoot growth and development. A relevant study has indicated that cell number is determined by cell division within the shoot apical meristem (SAM), and these cells are enlarged after leaving the meristems^14^. Plant hormones play important roles in cell division and elongation, which determine the final plant architecture^12,15^. Studies in *Arabidopsis* and certain crops suggested that auxin and cytokinin are important phytohormones that interact to control the formation and maintenance of the SAM, and they are linked to the outgrowth of axillary meristems, which drive shoot growth^3,16–18^. Numerous studies have also emphasized the dominant role of phytohormones in regulating the plant architecture of woody plants. Studies on columnar apple trees revealed that IAA (indole-3-acetic acid) and GA (gibberellic acid) are important hormones correlated with columnar habit^19^. High levels of GA increased the plant height of pine trees^20^, and the dwarf traits (*dw*) of peach were reportedly regulated by the GA receptor *GID1c*^6^. Although breeding efforts have been implemented to cultivate different tree sizes in many tree crops, the underlying molecular mechanisms remain unknown. Moreover, the mechanisms underlying the effect of phytohormones on the cell number and cell elongation and the subsequent impacts of these changes on the plant architecture of woody plants remain poorly understood. Therefore, additional studies on the mechanisms that control tree growth habits are required because the findings may provide a theoretical basis for other plant species.

*Lagerstroemia* has diverse plant forms from dwarf miniature shrubs to large trees, and it represents a suitable model for investigating the plant architecture of woody plants. These plants have a large quantity of flowers and fruits; thus, large progeny populations can be obtained, and the seedlings generally grow quickly and bloom in a single year. These characteristics have led to the widespread use of *Lagerstroemia* in gardens, and this plant is regarded as an indispensable source of income for companies and nursery growers^21^.

Studies on the genetic control of *Lagerstroemia* plant architecture have been published in recent years^22,23^; however, genes involved in the regulation of plant architecture are poorly understood. RNA-seq technology enables the analysis of whole transcriptomes. Based on the analysis of the transcriptional profiles between dwarf and non-dwarf progenies in the present research, potential target genes have been identified to reveal morphophysiological changes, including changes in the phenotype, anatomy and endogenous hormone levels. The data revealed the pivotal role of auxin in regulating *Lagerstroemia* plant architecture, suggested regulatory relationships between auxin and cell patterning, and provided insights into the related candidate genes at the transcriptional level. These findings will provide a foundation for further functional genomic studies to improve woody plant architecture.

## Results

### Phenotypic characterization and statistical analysis

**Figure 1:**
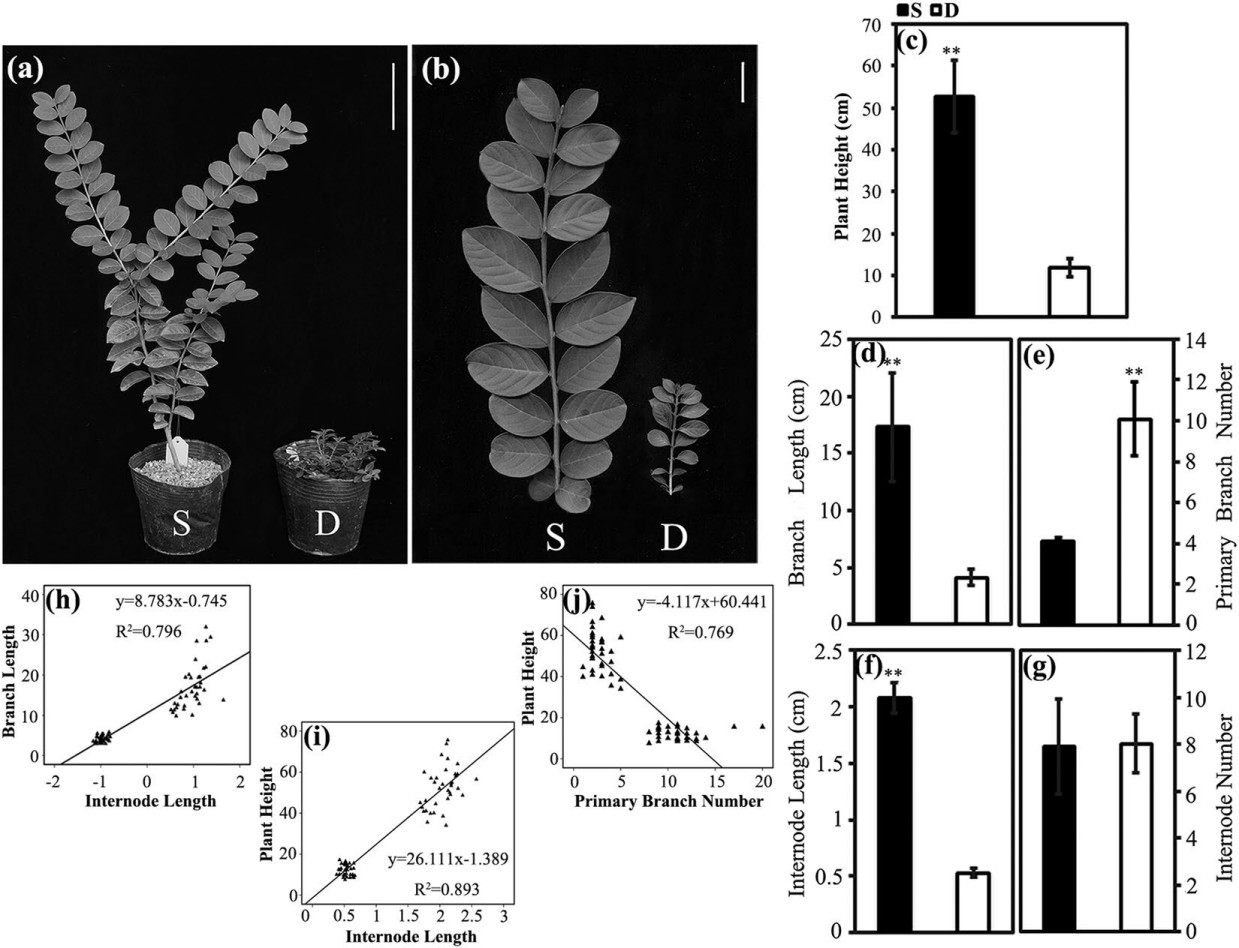
Phenotypic characterization of non-dwarf (S) and dwarf (D) progenies. (**a**) Plant architecture of non-dwarf and dwarf progenies. (**b**) Branches of non-dwarf and dwarf progenies. (c) Plant height of non-dwarf and dwarf progenies. (**d**) Branch length of non-dwarf and dwarf progenies. (**e**) Primary branch number of non-dwarf and dwarf progenies. (**f**) Internode length of non-dwarf and dwarf progenies. (**g**) Internode number of non-dwarf and dwarf progenies. Relationships between branch length and internode length (**h**), between plant height and internode length (**i**), and between plant height and primary branch number (**j**) on 36 non-dwarf plants and 36 dwarf plants. Each data represents a mean ± SD of three biological replicates. Student’s *t*-test was used to assess the significant differences, and the asterisk ‘**’ indicates significance level at P < 0.01. Bar = 10 cm in (**a**), 2 cm in (**b**).

According to the phenotypic data, the dwarf progenies were characterized by short branches and prolific branching (Fig. 1a,b). We measured five phenotypic traits, and the descriptive statistics of these plant architecture traits were presented in Supplementary Table S1. Compared with the non-dwarf progenies, the dwarf individuals displayed significantly decreased plant heights (P-value = 0.001, Fig. 1c) and branch (P-value = 0.009, Fig. 1d) and internode lengths (P-value = 0.000037, Fig. 1f) and significantly increased primary branch numbers (P-value = 0.005, Fig. 1e). No differences were detected in the internode number between the dwarf and non-dwarf progenies (Fig. 1g). As the branch length of *Lagerstroemia* was analysed from two aspects, including the internode length and internode number, the different branch lengths were mainly attributed to different internode lengths instead of internode numbers. Further linear regression analyses indicated that the internode length could primarily explain the branch length (R^2^ = 0.796, Fig. 1h) and plant height (R^2^ = 0.893, Fig. 1i). The primary branch number was also important for explaining the plant height (R^2^ = 0.769, Fig. 1j).

### Relative contribution of cell division and cell elongation to internode length

**Figure 2:**
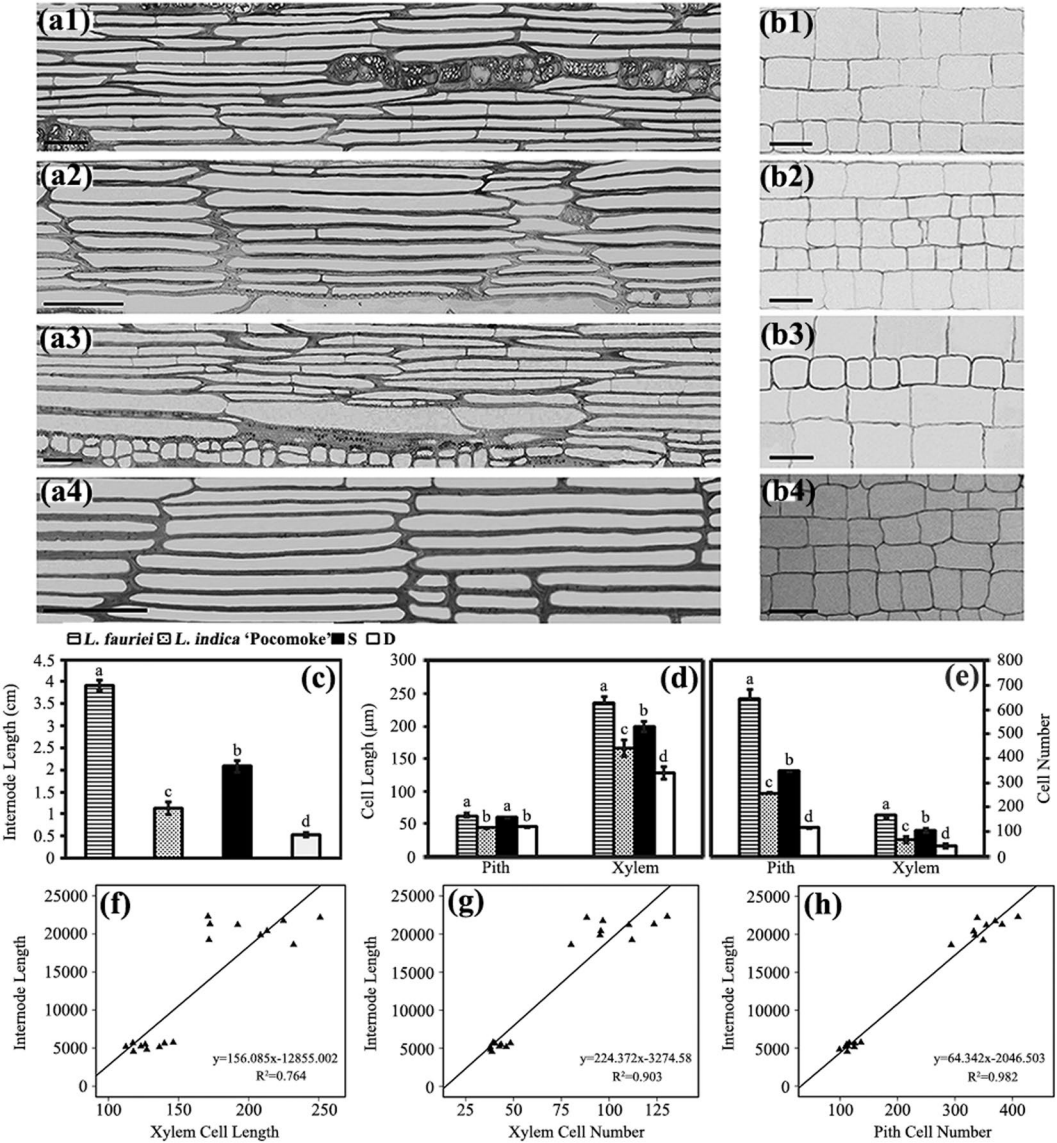
Analysis on longitudinal sections of xylem and pith of parents and progenies. Longitudinal sections of xylem: (a1) *L. fauriei*; (a2) *L.indica* ‘Pocomoke’; (a3) non-dwarf progenies; (a4) dwarf progenies. Longitudinal sections of pith: (b1) *L. fauriei*; (b2) *L.indica* ‘Pocomoke’; (b3) non-dwarf progenies; (b4) dwarf progenies. (**c**) Internode length of parents and progenies. (**d**) Pith cell length and xylem cell length of parents and progenies. (**e**) Pith cell number and xylem cell number of parents and progenies. Error bars represent mean ± SD. Different letters on the column indicate significance level at P < 0.01. Bar = 50 μm in (**a,b**). Relationships between internode length and xylem cell length (**f**), and between internode length and xylem cell number (**g**) were estimated in xylems of internodes of nine non-dwarf and nine dwarf progenies. Relationship between internode length and pith cell number (**h**) was estimated in piths of internodes on nine non-dwarf and nine dwarf progenies.

The cell number and cell length are primary cell characteristics that contribute to internode length. These parameters were investigated in the xylem and pith of parents and progenies (Fig. 2a,b). Analysis of the anatomy showed that the variations of cell length in the pith could not explain the variations of internode length in progenies and parents, whereas the xylem cell length showed significant differences, which could explain the internode length variations in the parents and progenies (R^2^ = 0.764; Fig. 2c,d,f). Regarding the cell number, highly significant differences were detected in both the xylem and pith of the parents and progenies, and the variations of cell number were consistent with the variations of internode length in the parents and progenies (Fig. 2c,e). Further linear regression analyses suggested that the cell number in the xylem and pith primarily indicated the internode length of parents and progenies (R^2^ showed 0.903 and 0.982, respectively; Fig. 2g,h).

### Endogenous hormone levels in SAMs and internodes

**Figure 3:**
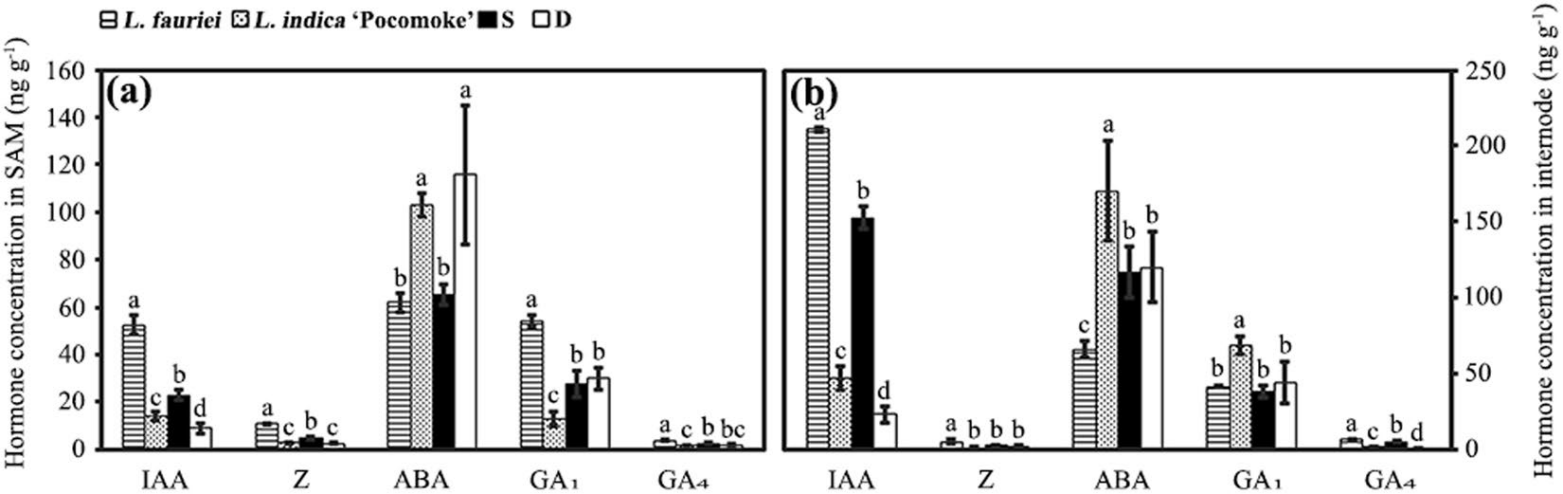
Hormone concentrations in SAMs (**a**) and internodes (**b**) of parents and progenies. Each value represents a mean ± SD of three biological replicates of which each includes three technical replicates. Statistical differences were assessed using one-way ANOVA. Different letters on the column indicate significance level at P < 0.05.

We compared the levels of IAA, zeatin (Z), abscisic acid (ABA), gibberellin A_1_ (GA_1_) and GA_4_ in the SAMs and internodes of the parents and progenies. Data analysis showed that the concentration of IAA in the SAMs of the parents and progenies (Fig. 3a) and the concentrations of IAA and GA_4_ in the internodes of the parents and progenies (Figs 3b and S1) showed highly significant differences (Table 1). These concentration variations could explain the variations in the cell number and xylem cell length of the parents and progenies (Figs 2d,e and 3), whereas the concentration variations of the other phytohormones in the SAMs and internodes of parents and progenies were not consistent with the histological and phenotypic variations in the parents and progenies.

**Table 1:** Endogenous hormones levels in the SAM and internode tissues of parents and progenies.

	Hormone concentration in SAM (ng g^−1^)				Hormone concentration in internode (ng g^−1^)			
	*L. fauriei*	*L. indica* ‘Pocomoke’	S	D	*L. fauriei*	*L. indica* ‘Pocomoke’	S	D
IAA	52.59 ± 4.22a	14.14 ± 1.81c	22.78 ± 2.06b	8.87 ± 2.19d	210.96 ± 1.13a	47.15 ± 8.14c	152.72 ± 7.69b	22.91 ± 5.08d
Z	10.71 ± 0.48a	2.78 ± 0.42c	4.87 ± 0.43b	2.57 ± 0.56c	4.96 ± 1.57a	1.20 ± 0.19b	2.23 ± 0.38b	1.93 ± 0.32b
ABA	61.99 ± 3.86b	102.9 ± 5.12a	65.13 ± 4.43b	115.68 ± 29.42a	66.13 ± 5.33c	170.46 ± 32.95a	116.64 ± 16.53b	120.25 ± 22.74b
GA_1_	54.17 ± 2.32a	12.84 ± 3.1c	27.59 ± 5.46b	29.83 ± 4.76b	41.39 ± 0.28b	68.86 ± 5.82a	38.43 ± 3.83b	43.95 ± 13.82b
GA_4_	3.80 ± 0.43a	1.44 ± 0.14c	2.55 ± 0.47b	1.91 ± 0.25bc	6.79 ± 0.32a	2.06 ± 0.03c	5.06 ± 0.21b	0.90 ± 0.10d

Each value represents a mean ± SD of three biological replicates of which each includes three technical replicates. Statistical differences were assessed using one-way ANOVA. Different letters in the same row indicate significance level at P < 0.05.

### Illumina sequencing

RNA was isolated from the SAMs collected from each clonal line of the dwarf and non-dwarf progenies, and three biological replicates of each plant-type progeny were performed. A total of six samples were sequenced, generating 346,716,654 clean reads representing 52 Gbp for further analyses (Supplementary Table S2). Because of the lack of genome sequences for *Lagerstroemia indica*, all clean reads were *de novo* assembled and the reference sequences were obtained. The length frequency distribution and the length distributions of the assembled transcripts and unigenes were presented in Supplementary Tables S3 and S4, respectively. The total mapped clean reads were more than 75% in the six samples (Supplementary Table S5).

The total unigenes were annotated with seven databases (Supplementary Table S6). Of the 93,161 unigenes, 54.16% of the unigenes were annotated in at least one database, with 49.3% and 35.53% of the total unigenes annotated in the Nr and SwissProt databases, respectively. According to the Gene Ontology (GO) database, 32,194 unigenes were annotated to 55 GO terms in level 2, which included 23 biological process categories, 18 cellular component categories and 14 molecular function categories (Supplementary Table S7). Among the 26 KOG (Clusters of Orthologous Groups of proteins) categories, ‘General function prediction only’ (2796) was the most frequent category, followed by ‘Posttranslational modification, protein turnover, chaperones’ (2,254) and ‘Signal transduction mechanisms’ (1405) (Supplementary Table S8). A total of 16,321 unigenes were annotated with the KO (KEGG Ortholog) database, and the most representative pathways were ‘Carbohydrate metabolism’ (1,713) and ‘Signal transport’ (1,678) (Supplementary Table S9).

### Comparative analysis of DEGs

**Figure 4:**
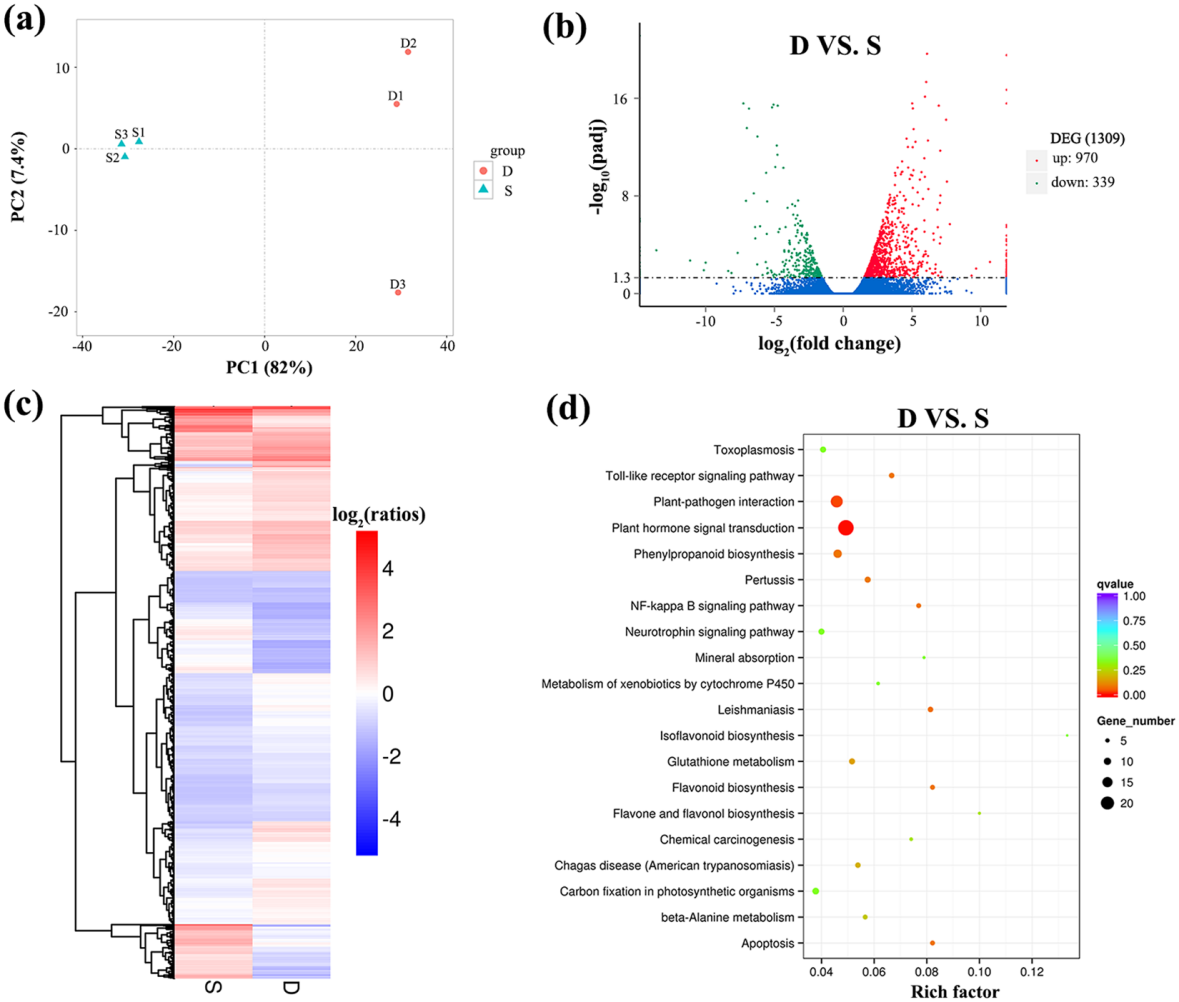
Analysis of differentially expressed genes (DEGs) in dwarf and non-dwarf progenies. (**a**) Principle component analysis (PCA) of the RNA-seq datasets. The blue triangles indicate the non-dwarf samples and the red circles indicate the dwarf samples. (**b**) Volcano plot of gene differential analysis of dwarf vs. non-dwarf (D VS. S). Filter of differential genes is padj <0.05. Every plot represents a gene. The blue circles indicate genes without significant difference, the red circles indicate up-regulated genes and the green circles indicate down-regulated genes. (**c**) Overall cluster analysis of DEGs in the transcriptomic comparisons of D vs. S. FPKM was used to estimate the level of gene expression. The color that changes from red (highly expressed) to blue (low expression) represents the relative expression level value log_2_ (ratios). (**d**) Top 20 KEGG pathway enrichment of up- and down-regulated DEGs. The number of genes in each pathway is equal to the dot size. The dot color represents the q-value. The smaller the q-value, the redder the dot. Rich factor = number of DEGs enriched in certain pathway term/ number of unigenes annotated in this pathway term. All up- and down-regulated DEGs are listed in detail in Supplementary Table S10.

A principal component analysis identified different plant architecture clusters, with the first component explaining 82% of the variance among the samples (Fig. 4a), which indicated considerable differences between groups and good repeatability within groups. In total, 1,309 genes were identified between the dwarf (D) and non-dwarf (S) progenies. Nine hundred and seventy up-regulated DEGs and 339 down-regulated DEGs were identified in the dwarf progenies compared with the non-dwarf progenies (Fig. 4b, Supplementary Table S10). The clustered patterns of all the DEGs were generated based on their fragments per kilobase of transcript per million base pairs sequenced (FPKM) values (Fig. 4c, Supplementary Table S11).

The functions of up- and down-regulated DEGs from the D vs. S comparison were evaluated using the GO database. The results suggested that only the term ‘carbohydrate binding’ in the molecular function category was enriched (Supplementary Fig. S2a). In the enrichment analysis of up-regulated DEGs, eight terms were enriched (Supplementary Fig. S2b). No term was enriched in the down-regulated genes (Supplementary Fig. S2c). All the DEGs were subjected to a KEGG pathway enrichment analysis. A total of 213 pathways were enriched, and the top 20 pathways were displayed in Fig. 4d. ‘Plant hormone signal transduction’ was the most enriched pathway, suggesting the highly important role of phytohormone in the plant architecture regulation.

### Identification of phytohormone-associated genes

**Figure 5:**
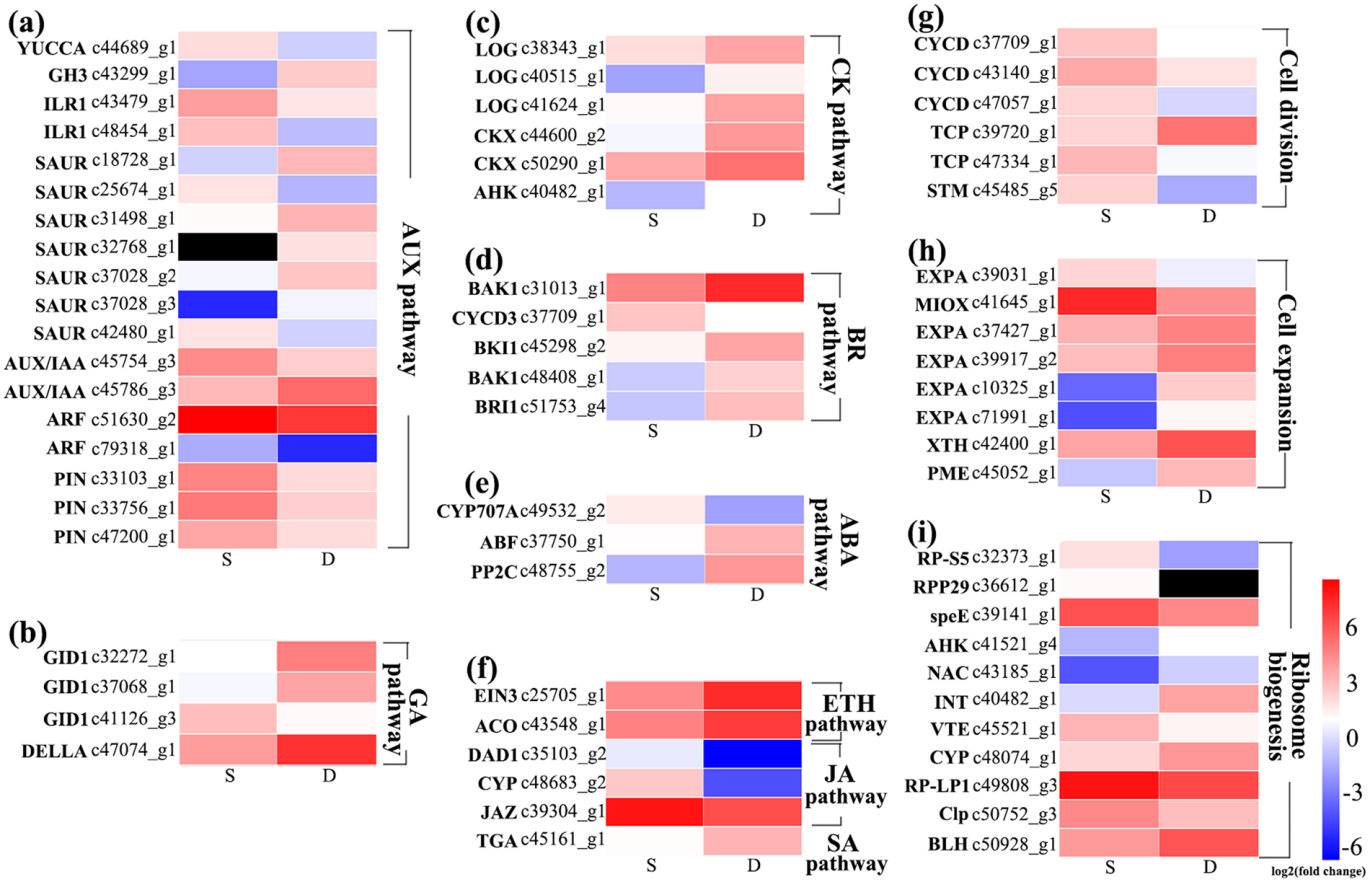
Heat maps of genes related to phytohormone pathways, cell division and cell expansion regulating the *Lagerstroemia* plant architecture. (**a**) Biosynthesis, transport and signalling of auxin (AUX). (**b**) Gibberellic acid (GA) signalling pathway. (**c**) Biosynthesis, degradation and signalling of cytokinin (CK). (**d**) Brassinosteroid (BR) signalling pathway. (**e**) Abscisic acid (ABA) metabolism and signalling pathways. (**f**) ethylene (ETH), jasmonic acid (JA), and salicylic acid (SA) pathways. (**g**) DEGs related to cell division. (**h**) DEGs related to cell expansion. (**i**) DEGs assigned to ‘ribosome biogenesis’ term regulating the cytoplasmic growth. Red and blue indicate up- and down-regulated transcripts, respectively, from the two comparisons (log2-fold change), while black indicates no expression. All genes are listed in detail in Supplementary Table S12.

We extracted genes associated with phytohormone biosynthesis, transport and signal transduction to further assess the status of phytohormones in the dwarf and non-dwarf progenies (Fig. 5 and Supplementary Table S12) and further combined these results with the above results to examine the hormone molecular networks.

A total of 18 DEGs annotated in auxin biosynthesis, homeostasis, transport and signalling were identified (Fig. 5a). *YUCCA6* (c44689_g1), encoding indole-3-pyruvate monooxygenase^24^, was down-regulated. The expression of GH3 (c43299_g1), encoding an IAA-amido synthetase^25^, was nearly four times higher than that in non-dwarf progenies. Two members of the ILR1-like family (c43479_g1 and c48454_g1), which encode IAA-amino acid hydrolases^26^, were down-regulated. Three unigenes (c33756_g1, c47200_g1, c33103_g1) annotated in the auxin efflux carrier family^27,28^ were significantly down-regulated in the dwarf progenies. In the auxin signalling pathway, members of the AUX/IAA family, auxin response factors (*ARF*s) and the small auxin-up RNA (*SAUR*) family were differentially expressed, with high levels of expression in *IAA26* (c45786_g3) and SAUR (c18728_g1, c37028_g3, c31498_g1, and c37028_g2). Two unigenes (c42480_g1, c25674_g1) annotated in the SAUR family proteins were down-regulated, and one unigene belonging to this protein family showed no expression in the non-dwarf samples. Two unigenes (c79318_g1, c51630_g2) annotated as ARF were both significantly down-regulated in the dwarf progenies.

Unigenes annotated in the GA signalling pathway were also differentially expressed (Fig. 5b). Three GA receptors^29^ (*GID*; c47074_g1, c32272_g1 and c37068_g1) showed a threefold increased expression in the dwarf progenies. One unigene (c41126_g3) belonging to the DELLA protein family^29^ was down-regulated. No DEG was annotated in the GA biosynthesis and metabolism pathways in the SAMs of the dwarf progenies. Unigenes that were annotated in the cytokinin biosynthesis and signalling pathways had higher expression in the dwarf progenies (Fig. 5c), and they included *LOG* (c40515_g1, c41624_g1, c38343_g1) in the cytokinin biosynthesis pathway^30^ and *CKX* (c44600_g2 and c50290_g1) of cytokinin dehydrogenase gene family^31^ and the cytokinin receptor (*AHK*; c40482_g1)^32^, which presented a nearly twofold higher expression in the dwarf progenies than in the non-dwarf progenies. The receptor of BR (*BRI1*; c51753_g4), BRI-associated kinase 1 (*BAK1*; c48408_g1 and c31013_g1) and BRI1 kinase inhibitor 1-like (*BKI1*; c45298_g2) from the BR signalling pathway^33,34^ showed up-regulated expression (Fig. 5d). The unigene (c37750_g1) encoding ABA 8′-hydroxylase (CYP707A) which regulates ABA catabolism^35^, showed more than threefold down-regulation in the dwarf progenies. Two unigenes, type 2C protein phosphatase (PP2C) and ABA responsive element binding factor (ABF)^35^, annotated in the ABA signalling pathway were significantly up-regulated (Fig. 5e). The transcription of ethylene (ETH), jasmonic acid (JA) and salicylic acid (SA) pathway genes also showed changes between the different plant architecture progenies (Fig. 5f).

### Cell-cycle and cell-growth-related genes

Sablowski and Dornelas (2014) reported that mitotic cycles regulate the cell number in plant tissues, and cytoplasmic growth and cell-wall extension are two determinants that affect the cell volume^36^. The D-type cyclin (CYCD) genes are key cell cycle regulators responsible for triggering G1 to S and G2 to M transitions^37^. Twenty-one unigenes were annotated as CYCD in the *Lagerstroemia* transcriptome. The unigenes c37709_g1, c47057_g1 and c43140_g1 which respectively showed high homology to cyclin-D3-1, cyclin-D5-1 and putative cyclin-D6-1 were all down-regulated in the dwarf progenies. TCP transcription factors have been implicated in the control of cell cycle-related genes in plants^38^. Two unigenes (c39720_g1 and c47334_g1) annotated in two distinct TCP sub-families were up- and down-regulated in the dwarf progenies, respectively. TCP proteins are also important regulators of shoot branching. A study on soybean indicated that a candidate gene encoding TCP transcription factor regulated branch outgrowth^39^. *SHOOT-MERISTEMLESS* (*STM*) was found to prevent cellular differentiation and endoreduplication and thus provided sustained mitotic activity to maintain the undifferentiated cells in the SAM^40^. Only one gene (c45485_g5) was annotated as *STM* in the transcriptome. The expression of c45485_g5 showed a fourfold decreased expression in the dwarf progenies (Fig. 5g, Supplementary Table S12).

Cell enlargement requires wall loosening, which is primarily regulated by expansins and endotransglycosylase/hydrolases (XTHs)^41^. Forty-nine putative members of the expansin family were detected in the *Lagerstroemia* samples. Four of these enzymes (c37427_g1, c39917_g2, c10325_g1, c71991_g1) were highly up-regulated, and the other enzyme (c39031_g1) was down-regulated in the dwarf progenies. A total of 47 unigenes were annotated as XTHs in the *Lagerstroemia* transcriptome, and only the expression of c42400_g1 was up-regulated in the dwarf progenies. Pectin plays an important role in the cell-wall stiffness definition^3,41^. One unigene (c45052_g1) annotated as pectin methylesterase (PME) was determined in the transcriptome and was markedly up-regulated in the dwarf progenies. Myo-inositol oxygenase (MIOX) was regarded as a useful tool for the manipulation of cell wall composition^42^. The expression of *MIOX2* (c41645_g1) was decreased more than threefold in the dwarf progenies (Fig. 5h, Supplementary Table S12).

The expression of unigenes annotated in ribosome biogenesis was also assessed because proper regulation of the process is mandatory for cellular growth and proliferation^43^. In the transcriptome, ten DEGs were assigned to ‘ribosome biogenesis’ (GO:0042254) under the ‘biological process’ category. Only one unigene (c36612_g1) annotated as *RPP29* was enriched in the ‘Ribosome biogenesis in eukaryotes’ term of the KEGG pathway. This gene showed no expression in the dwarf progenies according to the transcriptome analysis (Fig. 5i, Supplementary Table S12).

### Validation of unigene expression

Twenty-two DEGs related to phytohormone pathways, cell cycle and cell growth were randomly selected to validate the accuracy and reproducibility of the transcriptome data. The results indicated that the relative levels of unigenes obtained via qRT-PCR were consistent with the values produced from the RNA-seq data (Supplementary Fig. S3, Supplementary Tables S11 and S13). The correlation coefficient was 0.839 for the data obtained by the two approaches for estimating the expression levels of the 22 transcripts (Supplementary Fig. S4).

### Responses of phenotypic traits to exogenous IAA and GA_4_

**Figure 6:**
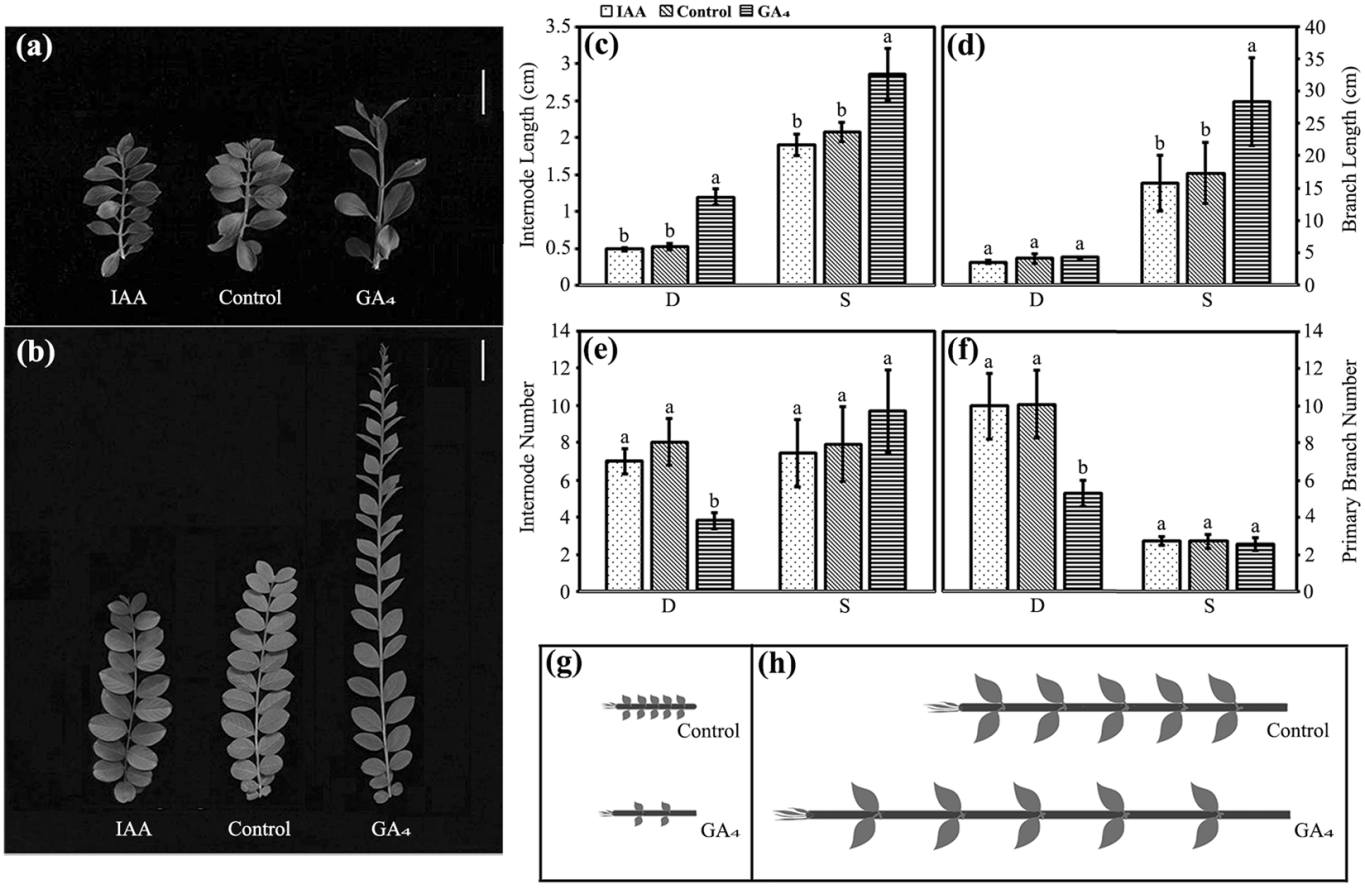
Phenotype variations of non-dwarf and dwarf progenies after exogenous IAA and GA_4_ treatments. Branches of dwarf (**a**) and non-dwarf (**b**) progenies treated with IAA and GA_4_, respectively. Bar = 2 cm in (**a**), 5 cm in (**b**). Variations of internode length (**c**), branch length (**d**), internode number (**e**) and primary branch number (**f**) of dwarf and non-dwarf progenies after treated with IAA and GA_4_. Model of pre- and post-treatment with GA_4_ on dwarf branches (**g**). Model of pre- and post-treatment with GA_4_ on non-dwarf branches (**h**). Plants without hormone treatments served as controls. Error bars represent mean ± SD. Different letters on the column in (**c**–**f**) indicate significance level at P < 0.01.

To further understand how IAA and GA_4_ regulation affect the plant architecture of *Lagerstroemia*, we applied their corresponding exogenous hormones to the dwarf and non-dwarf progenies. Significant differences were not observed between the pre- and post-IAA treatment in either the dwarf progenies or the non-dwarf progenies (Fig. 6a–f). However, the GA_4_ treatment promoted internode elongation in the non-dwarf progenies, which resulted in a mean 1.6-fold increase in branch length (Fig. 6b–d). Significant effects were not observed on either the internode number or the primary branch number of the non-dwarf progenies (Fig. 6e,f). Intriguingly, notable differences were not detected in the branch length between dwarf progenies with and without the GA_4_ treatment (Fig. 6a,d). However, a mean 2.3-fold increase in the internode length (Fig. 6c) and a decrease in the internode number (Fig. 6e) were detected. Although the internode length of the dwarf progenies was obviously increased after the GA_4_ treatment, it was still significantly shorter than that of the untreated non-dwarf progenies. Additionally, the GA_4_ treatment inhibited the outgrowth of lateral buds in the dwarf progenies, which led to a decrease in the primary branch number (Fig. 6f). The shoot variations in the dwarf and non-dwarf progenies after exogenous GA_4_ application were displayed in Fig. 6g,h, respectively.

### Effects of exogenous GA_4_ on the histology of internodes

**Figure 7:**
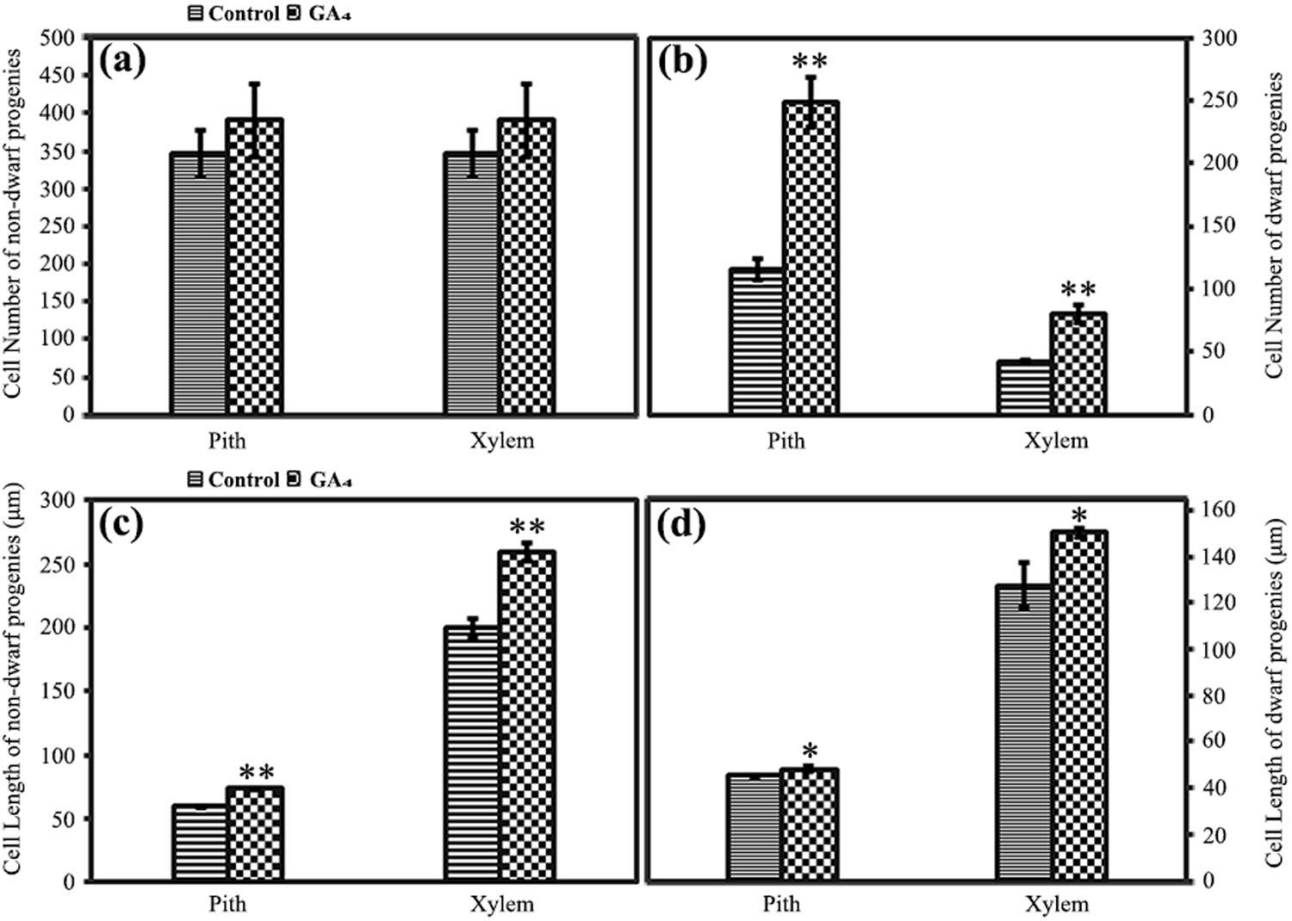
Histological observation of internodes of non-dwarf and dwarf progenies after exogenous GA_4_ application. Cell number variations in pith and xylem of non-dwarf (**a**) and dwarf (**b**) progenies after exogenous GA_4_ application. Cell length variations in pith and xylem of non-dwarf (**c**) and dwarf (**d**) progenies after exogenous GA_4_ application. Plants without hormone treatments served as controls. Error bars represent mean ± SD. Statistical differences were assessed using Student’s *t*-test. Asterisks ‘*’ and ‘**’ indicate significance levels at P < 0.05 and P < 0.01, respectively.

To elucidate the different phenotypic variations of dwarf and non-dwarf progenies after the GA_4_ treatment, histological variations of the internodes were examined. The results indicated that the cell number in the non-dwarf progenies was not affected by the exogenous GA_4_ treatment (Fig. 7a), whereas the cell numbers in both the pith (increased 115%) and the xylem (increased 92%) of the dwarf progenies were markedly increased compared with that of the controls (Fig. 7b). The cell lengths in the pith (increased 24%) and xylem (increased 30%) of the non-dwarf progenies were significantly increased after GA_4_ treatment compared with that of the untreated progenies (Fig. 7c), whereas the corresponding cell length in the dwarf progenies presented less dramatic increases (Fig. 7d).

### Effects of exogenous GA_4_ on the expression of candidate genes

DEGs involved in the regulation of auxin biosynthesis, transport and signalling pathways as well as the cell division were identified to explore the effects of exogenous GA_4_ on the gene expression in the dwarf progenies. The expression of candidate unigenes was analysed via qRT-PCR at 1 and 3 hours after the exogenous GA_4_ treatment. The expression of *YUCCA* unigene (c44689_g1) and *ARF* unigene (c51630_g2), which were assigned to the IAA biosynthesis and signalling pathways, respectively, was significantly increased within 1 hour (Supplementary Fig. S5a,b). Three *PIN* unigenes (c33756, c33103 and c47200) were also markedly increased within 1 hour post-treatment (Supplementary Fig. S5c–e). The expression of three *CYCD* unigenes (c37709, c43140 and c47057) regulating cell division continuously increased over 3 hours (Supplementary Fig. S5f–h). The increased expression of the *YUCCA* unigene and two *PIN* genes (c33756, c33103) was also apparent at 3 hours (Supplementary Fig. S5a–c).

## Discussion

A better understanding of the molecular mechanism underlying plant architecture can help accelerating the molecular breeding process and improve plant architecture to satisfy different needs. Essentially, the internode length of *Lagerstroemia* refers to the distance between adjacent pairs of nodes originating from cells located in the peripheral zone surrounding the SAM. Here, we found that the dwarf progenies possessed shorter and denser branches than did the non-dwarf progenies, and the short branch length was determined by the reduced internode length instead of the internode number. Anatomy analysis showed that the reduced internode length resulted from a decrease in the cell number of pith and xylem and a reduction in the elongation capability of xylem cells. A previous study has indicated that new cells are formed within the meristems, and these cells are enlarged after leaving the meristems^14^. Hence, the cell number devoted to the longitudinal sections of the internode was determined by cell division within the SAM, and the elongation of these cells occurred after leaving the SAM. Further investigation of our research indicated that the levels of auxin in the SAMs between dwarf and non-dwarf crapemyrtles showed highly significant differences, and the auxin levels in the internode between them also showed highly significant differences. Relevant studies have shown that auxin promotes cell division and meristem maintenance and also plays an important role in the establishment of cellular patterning^3,14^. In conclusion, we speculated that auxin played important roles in the SAM morphogenesis and establishment of cellular patterning of *Lagerstroemia*, which determined the branch length.

Based on the analyses on phenotype, histology and phytohormone metabolite levels, the DEGs involved in the regulation of phytohormone pathways as well as the cell number and cell length were identified to explore the underlying regulatory mechanism of plant architecture.

Auxin, cytokinin, GA and ABA are all important phytohormones in regulating plant growth and development. These phytohormones play important roles in cell proliferation and cell elongation^13^. The hormone analysis in the present study elucidated the important roles of IAA in the SAM and internodes of *Lagerstroemia* with different plant architecture. Based on the apical dominance theory^44^, the reduced IAA in the SAM might eliminate the apical dominance of the dwarf progenies, representing one of the important reasons for increased branching. To date, auxin has been designated as a plant morphogen. The concentration gradient of auxin, which is established by auxin biosynthesis, metabolism, and transport, is considered a driving force for organogenesis and patterning. Transcriptome analysis displayed the differential expression of genes in the auxin pathway and revealed the reason for different IAA levels in dwarf and non-dwarf crapemyrtles at the molecular level for the first time. The decreased expression of the *YUCCA6* (c44689_g1) in the auxin biosynthesis pathway, two members of the ILR1-like family (c48454_g1, c43479_g1) that can hydrolyse IAA-amido to free IAA and the increased expression of one member of the GH3 family (c43299_g1) that encodes the auxin-conjugating enzyme together resulted in reduced IAA levels in the dwarf samples. Previous studies have reported that the *PIN1* gene plays a major role in establishing gradients of auxin within developing organs^27^ and showed that *PIN6* and *PIN1C* might work together to play a role in the intracellular regulation of auxin homeostasis^27,28^. In the transcriptome, the unigenes c33756_g1, c47200_g1 and c33103_g1 showed high homology to *PIN1*, endoplasmic reticulum (ER)-localized *PIN6* and the PIN-LIKE auxin efflux carrier *PIN1C*, respectively, and down-regulated expression was observed in the dwarf progenies, which suggested abnormal auxin transport in the dwarf progenies. In the present transcriptomics analysis, the auxin-responsive protein gene (c45786_g3), which is a negative regulatory factor in auxin signalling, was up-regulated in the dwarf progenies. This gene can bind to and inhibit the functions of *ARF*s (c51630_g2 and c79318_g1)^45^ in the signalling pathway. Interestingly, the dwarf progenies exhibited insensitivity to exogenous IAA application (Fig. 6a), which might indicate that auxin biosynthesis and metabolism were not determinants of *Lagerstroemia* plant architecture, and insufficient auxin transport or abnormal signalling resulted in a lack of response. The transcription levels of the genes involved in the auxin transport and signalling pathways in *Lagerstroemia* plant architecture require further study. Previous studies have indicated that IAA is a messenger compound from the SAM that activates GA biosynthesis in the elongating internodes^46^. The decreased GA_4_ level in the internodes of dwarf progenies might be correlated with the down-regulated genes in the auxin pathway.

The growth and development of whole plants depend on both cell division and cell elongation. The D-type cyclin (CYCD) genes have consistently been considered transducers of external conditions, and they integrate hormone signals to inform the cells entering cell cycles^47^. In the transcriptome, all the DEGs annotated as CYCD showed down-regulated expression in the dwarf progenies, suggesting the important roles of *CYCD* genes in regulating *Lagerstroemia* cell division. Previous studies in model plants, such as *Arabidopsis* and poplar, have reported that the expression of certain *CYCD* genes could be regulated by auxin^48,49^, and auxin-responsive elements were also detected in the promoter regions of various cyclins^18^. Based on the transcriptome, we speculated that the reduced expression of auxin-responsive genes, including *AUX/IAA*, *ARF* and *CYCD* genes, inhibited the progression of the cell cycle, which resulted in a decreased cell number in dwarf *Lagerstroemia*. Studies in tobacco and *Arabidopsis* also suggested that the regulation of auxin response genes might be necessary for cell-cycle progression^18^. Previous studies have revealed that the plant-specific TCP family is correlated with the regulation of cell proliferation^38^, and two distinct TCP sub-families were suggested to act antagonistically, with class I genes promoting cell division and growth and class II genes inhibiting these parameters^50^. The function of *TCP* genes in *Lagerstroemia* development is unclear. The present data indicated that both the down-regulated TCP gene of class I (*TCP14*) and the up-regulated TCP gene of class II (*TCP12*) might play negative roles in cell division during the development of the dwarf *Lagerstroemia*. *TCP14* has been indicated to promote cell proliferation and modulate plant architecture in *Arabidopsis*, and the expression of genes related to cell division was also decreased in the *tcp14* mutant^38^. Studies on *TCP14* from upland cotton demonstrated its ability to regulate the expression of auxin response and transporter genes^51^, which suggested that a regulatory relationship might also exist between *TCP* genes and auxin response and transporter genes, and might affect the progression of the cell cycle. Further research is needed to examine this hypothesis.

Expansin proteins, which break hydrogen bonds between wall polymers, can cause cell-wall loosening to promote cell enlargement^41^. Interestingly, most expansin proteins were up-regulated in the dwarf samples, which suggested normal transcription and activity of the expansins. Previous studies have shown that transgenic plants that over-expressed an expansin isoform also exhibited impaired growth, with shorter leaves and internodes, because of a reduced susceptibility of cell walls to expansin action^52^. These data caused us to speculate that decreases in the cell wall in response to expansins represented the dominant restriction on cell enlargement in *Lagerstroemia*. Numerous studies in dicotyledonous plants, such as potato^53^ and tomato^54^, showed that the transcription of expansin genes was positively regulated by auxin. However, decreased IAA levels in the dwarf progenies did not lead to a decline in the transcription of expansins, suggesting that GA might play a principle role in regulating expansin activity, which was similar to the role of GA in poplar^55^. Moreover, the decreased susceptibility to expansin in the cell wall might primarily be affected by auxin in woody plants.

Although both the non-dwarf and dwarf progenies responded to exogenous GA_4_ application, they displayed entirely different phenotypic and anatomical variations compared with their controls. The cell number and internode number of the GA_4_-induced non-dwarf progenies did not show significant differences compared with those of the untreated non-dwarf progenies, whereas the cell length was significantly increased, which led to an elongated internode length after the GA_4_ treatment. Both the cell number and the cell length of GA_4_-induced dwarf progenies were markedly increased compared with those of the untreated dwarf progenies, which led to increased internode length, while the increased internode length of the dwarf progenies was not restored to that of the non-dwarf progenies.

Auxin-GA_4_ interaction might play an important role in the cell elongation of the internodes of *Lagerstroemia*. The cell length in the internodes of dwarf progenies after exogenous GA_4_ treatment was still significantly lower than that of the untreated non-dwarf progenies. Hormone analysis showed that the levels of GA_4_ and IAA in the internodes of the dwarf progenies were significantly lower than those of the non-dwarf progenies. A previous study showed that auxin might be required for the GA response^46^. Based on studies of the interactions between growth hormones in the stems of pea plants^56^, we speculated that the lower level of auxin in the internodes of dwarf progenies might accelerate the deactivation of GA, thereby presenting smaller increases in the internodes of dwarf progenies after treatment with equivalent exogenous GA_4_ in dwarf and non-dwarf progenies.

GA-dependent GIDs might partly rescue the defective auxin pathway, thereby increasing the cell number within the SAM of the dwarf progenies. The cell number within the SAM of the GA_4_-induced non-dwarf progenies did not show significant difference, while the cell number within the SAM of the dwarf progenies after the exogenous GA_4_ treatment was significantly increased compared with those of the untreated dwarf progenies. Hormone analysis indicated that the levels of GA_1_ in the SAMs of dwarf and non-dwarf progenies were not significantly different, and the GA_4_ levels in the SAMs of dwarf and non-dwarf progenies also showed no significant differences. Although the expression of GA receptor (*GID*) was up-regulated in the dwarf progenies, the capacity for cell division within the SAM was low. After the exogenous GA_4_ treatment, the expression of *YUCCA* in the auxin biosynthesis pathway, three *PIN* transporters, and *ARF* in the auxin signalling pathway in the SAM of the dwarf progenies were all increased. The expression of the three *CYCD* genes was also increased. These results suggested that the GA-dependent GID1-DELLA perception system might be established after GA treatment, and might promote the auxin pathway. This finding is consistent with the conclusions of Wang and Deng^57^.

Moreover, the up-regulated *GID* in the dwarf progenies might be associated with lateral bud outgrowth. Strigolactones (SLs) are a group of phytohormones that regulate shoot-branching outgrowth. In 2017, three articles proposed that SLs and GAs are interactional^58–60^. Studies on rice have shown that the negative regulation of SL biosynthesis by GA was dependent on the GA receptor *GID1*^60^. Further studies should explore the role of GA signalling in the control of shoot branching in *Lagerstroemia*. Additionally, leaves around the stems are developed from the leaf primordia within SAM and determine the internode number. The decreased number of primordia after treatment resulted in a decreased internode number in the dwarf progenies. Previous studies have shown that changes in the polarity of PIN localization redirect auxin fluxes to create the local auxin gradients required for primordia establishment^3^. Membrane dynamics are important for the polar localization of PINs that localize to plasma membranes^61,62^. The cell wall was also suggested to restrict the lateral diffusion of PINs, thereby contributing to the polar localization of PINs^63^. Exogenous GA_4_ changed the cell number and cell length of the dwarf progenies, which might lead to non-polar localization of the PINs that affected the initiation of lateral organs. Further studies are needed to examine this hypothesis.

The integrated analysis on phenotype, anatomy and phytohormone metabolite levels determined important hormones affecting the plant architecture of *Lagerstroemia*. Putative target genes on auxin pathway and cellular patterning were examined to explore the underlying regulatory mechanism of plant architecture. The present research will help to develop a better understanding of the mechanisms regulating the plant architecture of *Lagerstroemia* and other woody plants and improve the efficiency of ornamental plant molecular breeding.

## Methods

### Plant materials

Dwarf and non-dwarf progenies of an F_1_ segregating population were generated from a cross between *Lagerstroemia fauriei* and *L. indica* ‘Pocomoke’, parental lines with erect and dwarf growth habits, respectively. Among the segregating populations, three extreme dwarf progenies and three extreme non-dwarf progenies were selected and propagated by cutting. Parental lines used as the control materials were also obtained by cutting propagation. All the materials were transplanted into pots (16 cm × 14 cm) and maintained in a greenhouse at the China National Engineering Research Center for Floriculture (Beijing) (40°17″N, 116°39″E) under a natural photoperiod (25 June–28 August 2015) with 29 ± 3/21 ± 2 °C (day/night) and 42 ± 7%/64 ± 5% relative humidity (day/night). Finally, eight clonal lines (clonal lines of the dwarf progenies numbered D1, D2 and D3; clonal lines of the non-dwarf progenies numbered S1, S2 and S3; and parental clonal lines numbered P1 and P2) were generated, and each line included no fewer than 120 individuals. Sixty individuals with consistent growth vigour were selected from each clonal line after one year. These one-year-old potted grown plants raised by cutting were uniformly pruned. When the buds immediately below the cut began to produce the first internode, the SAMs freed from the leaf, petiole and lower shoot tissues were collected for hormone content measurement and transcriptome determination. The SAMs of the sixty individuals in the same clonal line were collected as one sample and immediately frozen in liquid nitrogen. This group served as one biological replicate. Three biological replicates were performed for each plant-type progeny. Additionally, the first elongated internodes of the removed lateral buds were collected for hormone content measurement. The internodes of the sixty individuals in the same clonal line were also collected as one sample that served as one biological replicate. Three biological replicates were performed for each plant-type progeny.

### Phenotypic data collection

After annual growth ceased, branches from three different orientations of individuals were selected to estimate the branch length, internode number and internode length, and the plant height and primary branch number were also measured. Student’s *t*-test was used to assess the significant differences, and linear regression analysis was conducted using the SPSS Statistics 20.0 program (SPSS, Chicago, IL, USA).

### Histological observation of internodes

The internodes were sampled from a corresponding location of the branches in the parents and progenies. The internodes were split along the median longitudinal line and cut into 5-mm-long blocks. The samples were fixed in precooled (0–4 °C) FAA (38% formaldehyde/glacial acetic acid/70% ethanol/glycerin 1:1:18:1) and then dehydrated, infiltrated, embedded, and stained^64^. The median longitudinal sections (1 μm) were cut on a microtome (EM UC7, Leica, Wetzlar, Germany) and imaged using a microscope (Zeiss Axio Scope A1, Zeiss, Jena, Germany) equipped with a digital camera (ProgRes C5, JENOPTIK, Jena, Germany). On each longitudinal section photograph, the lengths of all the visible cells in the pith and xylem were determined by drawing a line across the cell to connect the walls, and the corresponding length was calculated using the software Nano Measurer (Fudan University, Shanghai, China). The total number of cells contributing to the longitudinal section of the internode was estimated by dividing the internode length by the mean cell length^10^. Three individuals in every clonal line were selected to serve as one biological replicate. Three biological replicates were performed in the dwarf and non-dwarf progenies. The parents used as controls were analysed together with the progenies, and comparisons of the cell length and number were performed via a one-way ANOVA. Linear regressions were performed between the internode length and cell length and between the internode length and cell number using the SPSS Statistics 20.0 program.

### Hormone content measurement

The SAMs and internodes collected from the progeny clonal lines numbered D1, D2, D3, S1, S2 and S3 were used for the measurements, and each sample was assayed using three technical replicates. For the P1 and P2 clonal lines, samples of three replicates from the SAMs and internodes were also collected for the hormone content measurements. The levels of IAA, Z, GA_1_, GA_4_ and ABA were detected using [^2^H_5_] IAA, [^2^H_5_] Z, [^2^H_2_] GA_1_, [^2^H_2_] GA_4_ and [^2^H_6_] ABA (OlChemIm s.r.o., Olomouc, Czech Republic) as internal standards, respectively. Identification and quantification of the five endogenous hormones were performed via high-performance liquid chromatography-mass spectrometry (QTRAP5500, AB Sciex, Redwood City, CA, USA), as previously described^65^.

### Transcriptome analysis

Three biological replicates of the dwarf (D1, D2 and D3) and non-dwarf progenies (S1, S2 and S3) were used, and the non-dwarf progenies served as control materials in the transcriptome analysis. RNA was extracted from the SAMs collected from each clonal line. A total of 3 µg of RNA for each sample was prepared for sequencing. Sequencing libraries were generated using NEBNext^®^ Ultra™ RNA Library Prep Kit for Illumina^®^ (NEB, USA). The clustering of the index-coded samples was performed on a cBot Cluster Generation System using TruSeq PE Cluster Kit v3-cBot-HS (Illumina). The Illumina HiSeq^TM^ 150 platform was used for sequencing. Clean reads with high quality were used for further analysis. Transcriptome assembly was accomplished using Trinity^66^. Gene functions were annotated using seven databases: Nr (NCBI non-redundant protein sequences, e-value = 1e^−5^), Nt (NCBI non-redundant nucleotide sequences, e-value = 1e^−5^), Swiss-Prot (manually annotated and reviewed protein sequence database, e-value = 1e^−5^), KOG/COG (e-value = 1e^−3^), Pfam (Protein family, e-value = 0.01), KO (e-value = 1e^−10^), and GO (e-value = 1e^−6^). FPKM values were used to estimate the levels of gene expression. Differential expression analyses were performed using the DESeq R package (1.10.1). Genes considered to be differentially expressed presented an adjusted P-value < 0.05. The GOseq R package-based Wallenius non-central hyper-geometric distribution^67^ was used to perform GO enrichment analyses of the DEGs. The KEGG Orthology-Based Annotation System (KOBAS)^68^ was used to test the statistical enrichment of the DEGs in KEGG pathways.

### RT-qPCR verification

To validate the levels of gene expression as reflected by the FPKM values, 22 transcripts were selected and subjected to RT-qPCR. The PrimeScript^TM^ RT Reagent Kit with gDNA Eraser (Takara Bio Inc., Shiga, Japan) was used to synthesize the first-strand cDNA from the total RNA. Each PCR reaction was set up in a 20-μl volume containing 2 μl of first-strand cDNA, 0.6 μl of gene-specific primers, 10 μl of SYBR Premix Ex Taq (Takara) and 7.4 μl of sterile distilled water. *EF-1α* (GenBank ID:MG704141) was used as the reference gene^69^. Each reaction included three biological replicates, which were analysed via PCR (CFX connect, Bio-Rad, USA), with an initial denaturation for 3 min at 95 °C, followed by 40 cycles of 5 s at 95 °C and 30 s at 60 °C. Melting curves were generated using the following programme: 95 °C for 15 s, 60 °C for 1 min, and 95 °C for 15 s. The 2^−ΔΔCt^ method was used to analyse the relative abundance of the selected transcripts.

### Exogenous application with IAA and GA_4_

One-year-old potted plants generated via cutting were selected, and the branches were uniformly clipped. When the bud immediately below the cut produced three expanded leaves, the branches were sprayed with equivalent distilled water, 50 mg l^−1^ IAA or 200 mg l^−1^ GA_4_ (BIODEE, Beijing, China) solution once every 7 days, which occurred 3 times in succession. Each plant-type material included three biological replicates, and each replicate included 12 individuals. Three weeks later, the branch length, internode length, internode number, and primary branch number were analysed. The internodes from the corresponding branch locations in the dwarf and non-dwarf progenies were sampled after treatment. The median longitudinal sections (1 μm) of internodes were prepared according to the method described above. The cell number and cell length of the pith and xylem were also counted according to the above method. The SAMs of the dwarf progenies were harvested at 1 and 3 hours after exogenous GA_4_ treatment, and plants sprayed with distilled water served as the controls (Time 0) to further explore the dynamic expression of candidate genes.

## Electronic supplementary material

Supplementary file

Supplementary Table S10

Supplementary Table S11

Supplementary Table S12

## Data Availability

All data generated or analysed during this study are included in this published article (and its Supplementary Information files).
