# Supplementary material for: Transcriptome analysis of the genes regulating phytohormone and cellular patterning in Lagerstroemia plant architecture
Source: Sci Rep. 2018 Oct 11;8:15162. doi: 10.1038/s41598-018-33506-8 (PMC6181930; doi:10.1038/s41598-018-33506-8)
Supplement: Supplementary file 1 — Supplementary file [file 41598_2018_33506_MOESM1_ESM.doc]

***Supplementary material:***

**Transcriptome analysis of the genes regulating phytohormone and cellular patterning in *Lagerstroemia* plant architecture**

Yiqian Ju^1^, Lu Feng^1^, Jiyang Wu^1^, Yuanjun Ye^1^, Tangchun Zheng^1^, Ming Cai^1^, Tangren Cheng^1^, Jia Wang^1^, Qixiang Zhang^1^, Huitang Pan^1^*

**1 SUPPLEMENTARY DATA**

Supplementary Figure S1. GO enrichment analysis.

Supplementary Figure S2. Comparison of expression profiles of 22 candidate genes measured by RNA-seq and qRT-PCR.

Supplementary Figure S3. qPCR validation of transcript levels evaluated by RNA-seq.

Supplementary Figure S4. Comparison of the relative expression of eight candidate genes as determined by qRT-PCR for 1 hour and 3 hours after exogenous GA_4_ treatment and compared with the control ones.

Supplementary Table S1. Descriptive statistics of five traits for *L. fauriei*, *L. indica* ‘Pocomoke’ and the dwarf (D) and non-dwarf (S) progenies.

Supplementary Table S2. The quality analysis of all reads from six samples.

Supplementary Table S3. Length frequency distribution of assembled transcripts and unigenes.

Supplementary Table S4. Length distribution of assembly transcripts and unigenes.

Supplementary Table S5. Number of total clean reads of the six samples mapped to reference sequences.

Supplementary Table S6. Success rate of unigenes annotation using seven databases.

Supplementary Table S7. GO annotation classification frequencies.

Supplementary Table S8. KOG annotation classification frequencies.

Supplementary Table S9. KO annotation classification frequencies.

Supplementary Table S10. Up- and down-regulated DEGs in the transcriptomic comparisons of D vs. S.

Supplementary Table S11. FPKM (fragments per kilobase of transcript per million base pairs sequenced) of the dwarf and non-dwarf samples.

Supplementary Table S12. Differentially expressed genes related to phytohormone pathway, cell division and cell expansion.

Supplementary Table S13. Forward and reverse primer sequences of genes evaluated using qPCR experiments.

**2 SUPPLEMENTARY FIGURES AND TABLES**

**2.1 Figures**


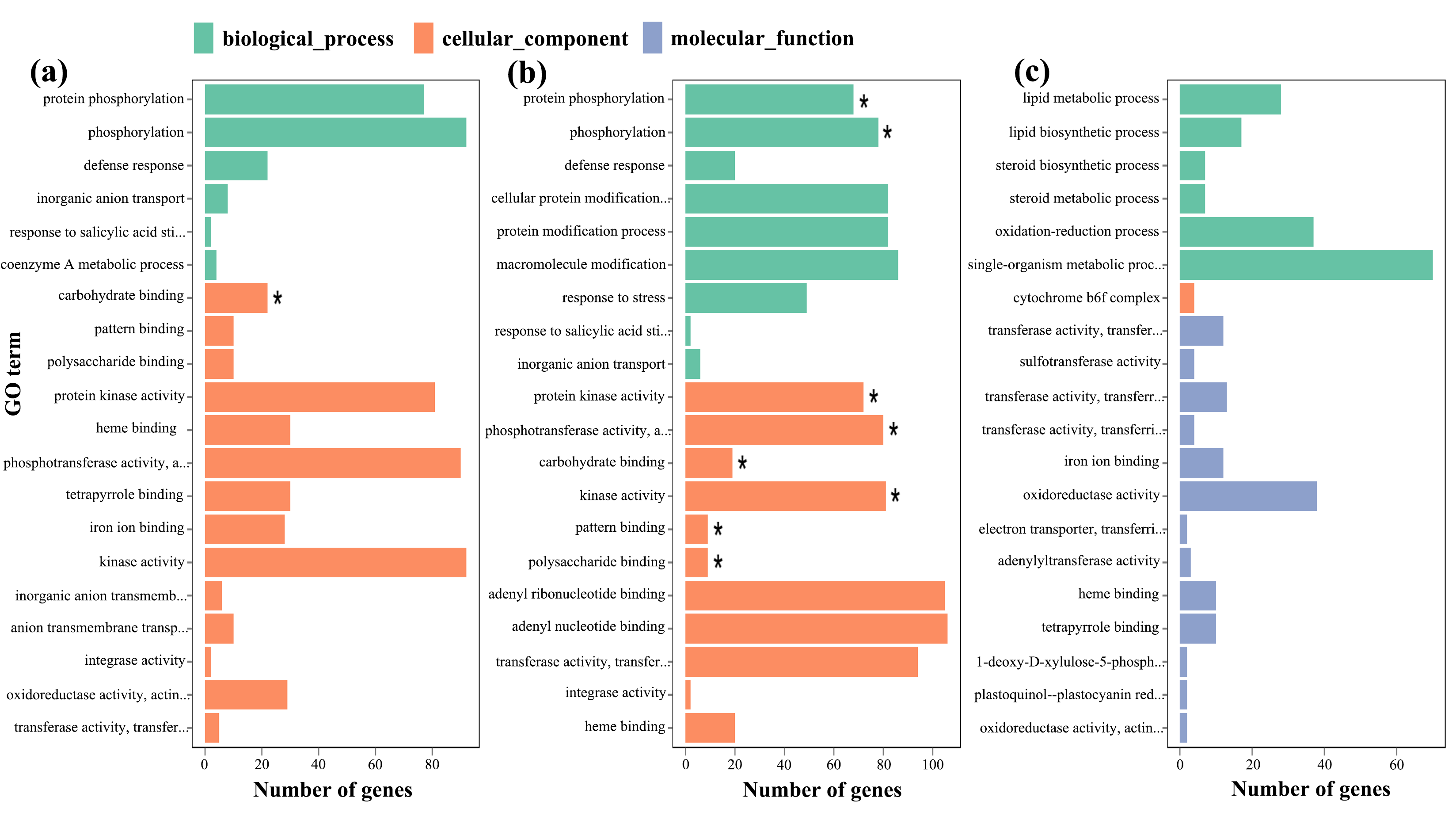


Figure S1. GO enrichment analysis. The top 20 GO enrichment analysis on all up- and down-regulated DEGs (a), up-regulated DEGs (b) and down-regulated DEGs (c) in the transcriptomic comparisons of D vs. S.


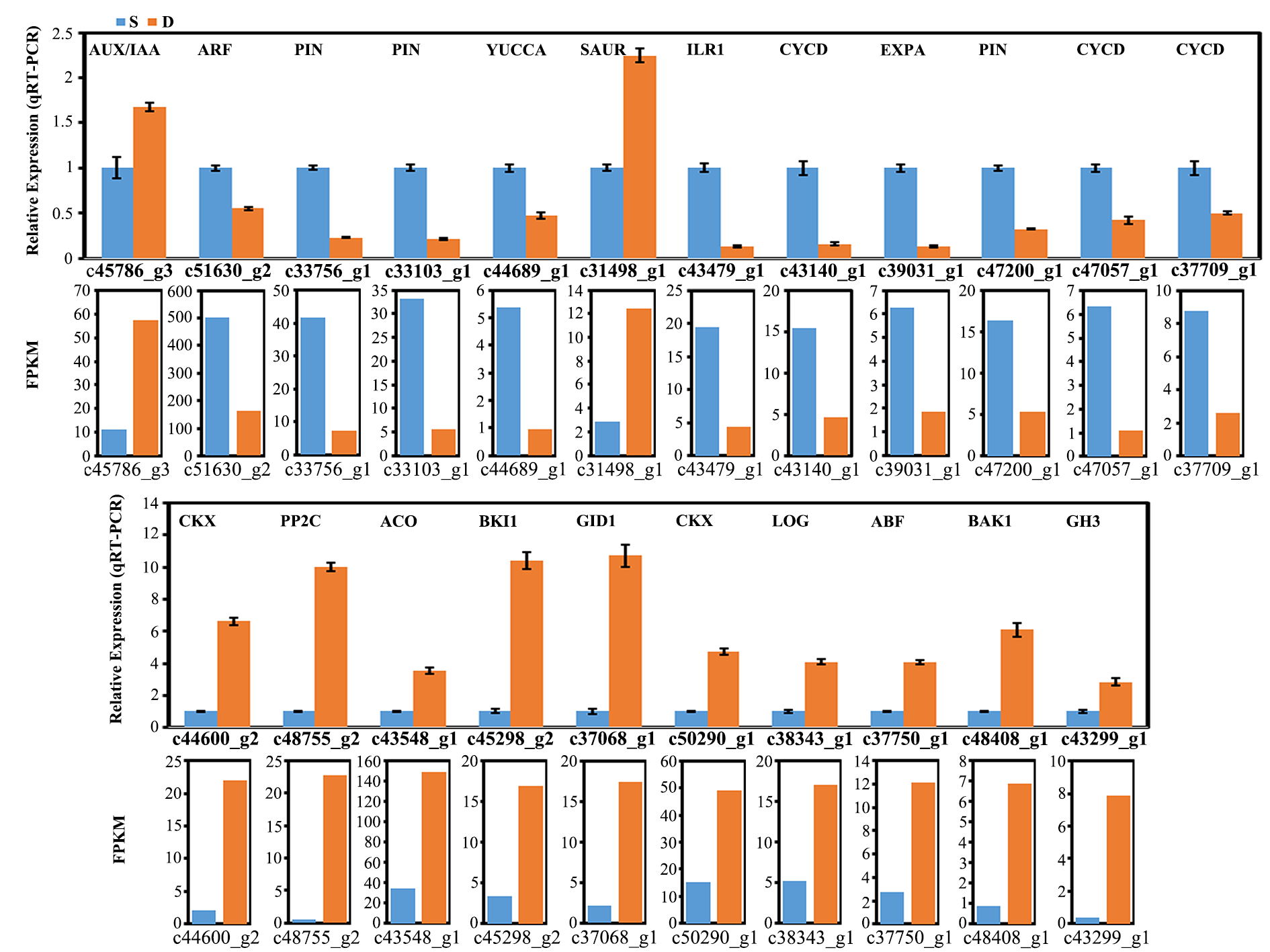


Figure S2. Comparison of expression profiles of 22 candidate genes measured by RNA-seq and qRT-PCR. Of them, 18 genes are assigned to different phytohormone pathways and the other 4 genes are related to cell cycle and cell growth. For qRT-PCR assay, the mean was calculated from three biological replicates each with three technical replicates. For RNA-seq, each column is the mean of three biological replicates. Error bars show SD.


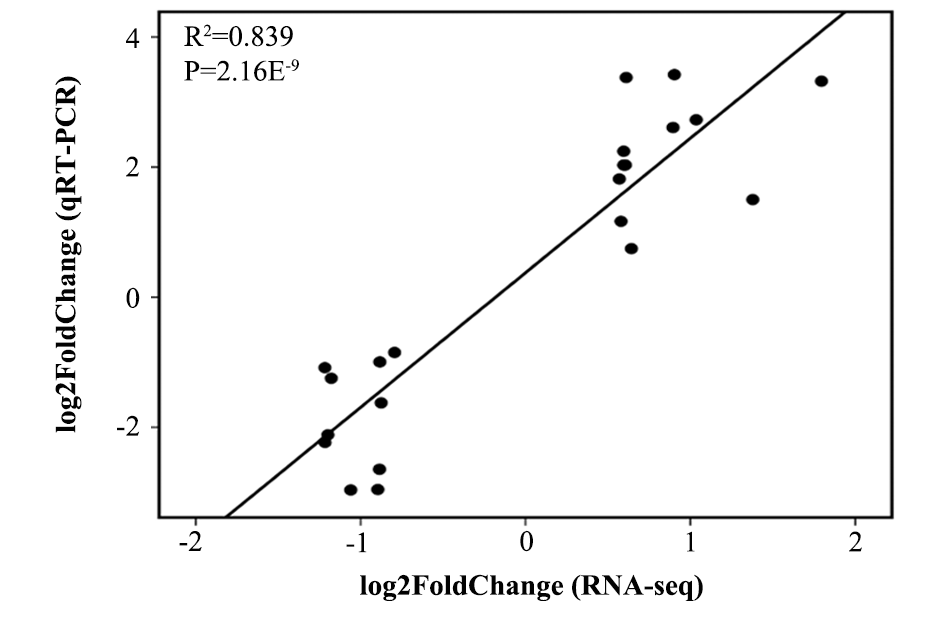


Figure S3. qPCR validation of transcript levels evaluated by RNA-seq. Correlation of the fold change and their associated P-values analyzed by RNA-seq platform with data obtained using qPCR in D vs. S. The information for each gene is shown in Supplementary Table S11 and S14. All qPCR data were collected from three biological replicates and there were three technical replicates for each sample. All standard errors within reasonable ranges.


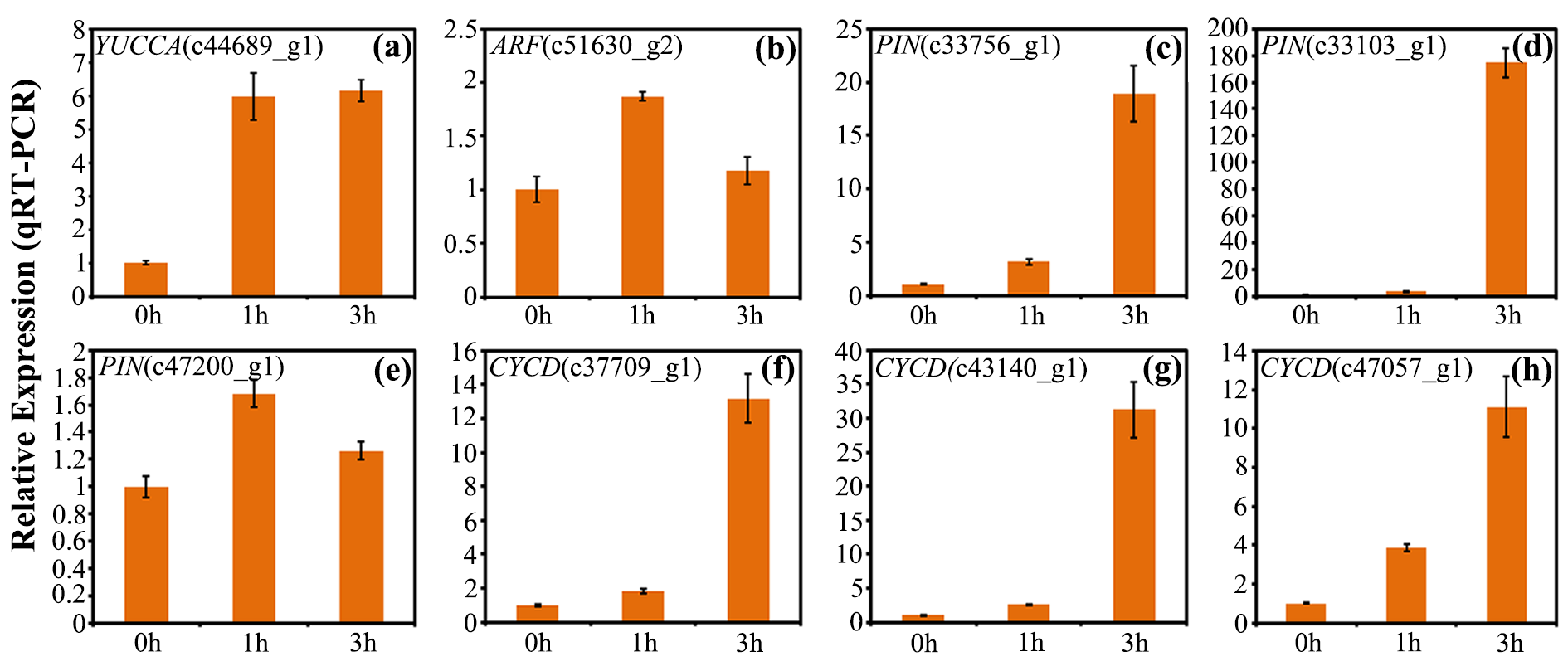


Figure S4. Comparison of the relative expression of eight candidate genes as determined by qRT-PCR for 1 hour and 3 hours after exogenous GA_4_ treatment and compared with the control ones. (a), *YUCCA* unigene (c44689_g1); (b), *ARF* unigene (c51630_g2); (c), *PIN* unigene (c33756); (d), *PIN* unigene (c33103); (e), *PIN* unigene (c47200); (f), *CYCD* unigene (c37709); (g), *CYCD* unigene (c43140); (h), *CYCD* unigene (c47057). The mean was calculated from three biological replicates each with three technical replicates. Error bars show SD.

**2.2 Tables**

Table S1. Descriptive statistics of five traits for L. fauriei, L. Indica ‘Pocomoke’ and their dwarf and standard progenies. ^x^ Mean ± SD. ^y^ Data followed by different letters within a column is statistically different (P<0.01).

|  | Plant Height^xy^ (cm) | Branch Length (cm) | Internode Length (cm) | Internode Number | Primary Branch Number |
| --- | --- | --- | --- | --- | --- |
| *L. fauriei* | 85.99±2.33a | 31.43±2.81a | 3.91±0.12a | 8±0.75a | 2±0.38d |
| *L. indica* ‘Pocomoke’ | 26.18±2.01c | 10.01±1.40c | 1.13±0.15d | 9±0.25a | 7±0.20b |
| S | 52.73±8.56b | 17.30±4.73b | 2.08±0.13b | 8±2.01a | 4±0.13c |
| D | 11.92±2.14d | 4.11±0.71d | 0.53±0.04d | 8±1.25a | 10±1.81a |

Table S2. The quality analysis of all reads from six samples. Error (%) represent the error rate of sequenced bases.

| Sample | Raw Reads | Clean Reads | Clean Bases | Error (%) | Q20 (%) | Q30 (%) | GC (%) |
| --- | --- | --- | --- | --- | --- | --- | --- |
| S1 | 77441304 | 54419772 | 8.16G | 0.02 | 97.27 | 91.27 | 49.51 |
| S2 | 89380062 | 61031450 | 9.15G | 0.02 | 97.12 | 90.88 | 49.56 |
| S3 | 84445162 | 57633676 | 8.65G | 0.02 | 97.05 | 90.69 | 49.68 |
| D1 | 66089482 | 53413062 | 8.01G | 0.03 | 96.01 | 88.4 | 50.25 |
| D2 | 69654414 | 57275302 | 8.59G | 0.02 | 96.44 | 89.51 | 50.32 |
| D3 | 89855412 | 62943392 | 9.44G | 0.02 | 97.28 | 90.98 | 49.33 |
| Total | 476865836 | 346716654 | 52.00G |  |  |  |  |

Table S3. Length frequency distribution of assembled transcripts and unigenes.

| Transcript length interval | 200-500bp | 500-1kbp | 1k-2kbp | >2kbp | Total |
| --- | --- | --- | --- | --- | --- |
| Number of transcripts | 68987 | 39850 | 44112 | 36034 | 188983 |
| Number of unigenes | 52751 | 17879 | 12024 | 10507 | 93161 |

Table S4. Length distribution of assembly transcripts and unigenes. N50 and N90 are defined as follows: the assembly transcripts are ordered from longest to shortest and accumulated, and N50 is the length of transcripts that are no less than 50% of the total length, N90 is the length of transcripts that are no less than 90% of the total length.

|  | Min Length | Mean Length | Median Length | Max Length | N50 | N90 | Total Nucleotides |
| --- | --- | --- | --- | --- | --- | --- | --- |
| transcripts | 201 | 1178 | 782 | 16400 | 1953 | 512 | 222668369 |
| unigenes | 201 | 836 | 424 | 16400 | 1601 | 308 | 77921920 |

Table S5. Number of total clean reads of the six samples mapped to reference sequences.

| Sample name | Total reads | Total mapped |
| --- | --- | --- |
| S1 | 54419772 | 40941954(75.23%) |
| S2 | 61031450 | 45925186(75.25%) |
| S3 | 57633676 | 43298702(75.13%) |
| D1 | 53413062 | 40523316(75.87%) |
| D2 | 57275302 | 43205958(75.44%) |
| D3 | 62943392 | 47580416(75.59%) |

Table S6. Success rate of unigenes annotation using seven databases. Nr-NCBI non-redundant protein sequences; Nt-NCBI non-redundant nucleotide sequences; KO-KEGG Ortholog database; SwissProt-A manually annotated and reviewed protein sequence database; Pfam-Protein family; GO-Gene Ontology; KOG-Clusters of Orthologous Groups of proteins.

|  | Number of Unigenes | Percentage (%) |
| --- | --- | --- |
| Annotated in NR | 45929 | 49.3 |
| Annotated in NT | 19540 | 20.97 |
| Annotated in KO | 16321 | 17.51 |
| Annotated in SwissProt | 33095 | 35.52 |
| Annotated in PFAM | 31648 | 33.97 |
| Annotated in GO | 32194 | 34.55 |
| Annotated in KOG | 16307 | 17.5 |
| Annotated in all Databases | 6025 | 6.46 |
| Annotated in at least one Database | 50461 | 54.16 |
| Total Unigenes | 93161 | 100 |

Table S7. GO annotation classification frequencies.

| GO ID | GO term (Lev2) | GO term (Lev1) | Gene number |
| --- | --- | --- | --- |
| GO:0032991 | macromolecular complex | Cellular Component | 6746 |
| GO:0043226 | organelle | Cellular Component | 6716 |
| GO:0044421 | extracellular region part | Cellular Component | 564 |
| GO:0055044 | symplast | Cellular Component | 6 |
| GO:0016020 | membrane | Cellular Component | 4802 |
| GO:0044456 | synapse part | Cellular Component | 15 |
| GO:0031974 | membrane-enclosed lumen | Cellular Component | 815 |
| GO:0005623 | cell | Cellular Component | 10203 |
| GO:0044425 | membrane part | Cellular Component | 4400 |
| GO:0044464 | cell part | Cellular Component | 10198 |
| GO:0031012 | extracellular matrix | Cellular Component | 220 |
| GO:0019012 | virion | Cellular Component | 664 |
| GO:0045202 | synapse | Cellular Component | 15 |
| GO:0005576 | extracellular region | Cellular Component | 636 |
| GO:0044423 | virion part | Cellular Component | 664 |
| GO:0030054 | cell junction | Cellular Component | 16 |
| GO:0044420 | extracellular matrix part | Cellular Component | 82 |
| GO:0044422 | organelle part | Cellular Component | 3625 |
| GO:0005488 | binding | Molecular Function | 18234 |
| GO:0030545 | receptor regulator activity | Molecular Function | 10 |
| GO:0003824 | catalytic activity | Molecular Function | 15068 |
| GO:0030234 | enzyme regulator activity | Molecular Function | 430 |
| GO:0016209 | antioxidant activity | Molecular Function | 170 |
| GO:0000988 | protein binding transcription factor activity | Molecular Function | 324 |
| GO:0005085 | guanyl-nucleotide exchange factor activity | Molecular Function | 50 |
| GO:0001071 | nucleic acid binding transcription factor activity | Molecular Function | 1169 |
| GO:0016530 | metallochaperone activity | Molecular Function | 3 |
| GO:0060089 | molecular transducer activity | Molecular Function | 467 |
| GO:0016247 | channel regulator activity | Molecular Function | 108 |
| GO:0005198 | structural molecule activity | Molecular Function | 1024 |
| GO:0004872 | receptor activity | Molecular Function | 249 |
| GO:0005215 | transporter activity | Molecular Function | 1992 |
| GO:0051179 | localization | Biological Process | 4862 |
| GO:0022414 | reproductive process | Biological Process | 236 |
| GO:0040007 | growth | Biological Process | 60 |
| GO:0044699 | single-organism process | Biological Process | 13603 |
| GO:0048511 | rhythmic process | Biological Process | 14 |
| GO:0001906 | cell killing | Biological Process | 37 |
| GO:0000003 | reproduction | Biological Process | 245 |
| GO:0050896 | response to stimulus | Biological Process | 3982 |
| GO:0071840 | cellular component organization or biogenesis | Biological Process | 2955 |
| GO:0051704 | multi-organism process | Biological Process | 1520 |
| GO:0032501 | multicellular organismal process | Biological Process | 664 |
| GO:0023052 | signaling | Biological Process | 2204 |
| GO:0022610 | biological adhesion | Biological Process | 213 |
| GO:0040011 | locomotion | Biological Process | 272 |
| GO:0050789 | regulation of biological process | Biological Process | 5602 |
| GO:0008152 | metabolic process | Biological Process | 17714 |
| GO:0048518 | positive regulation of biological process | Biological Process | 305 |
| GO:0044848 | biological phase | Biological Process | 18 |
| GO:0048519 | negative regulation of biological process | Biological Process | 246 |
| GO:0065007 | biological regulation | Biological Process | 6029 |
| GO:0002376 | immune system process | Biological Process | 142 |
| GO:0032502 | developmental process | Biological Process | 496 |
| GO:0009987 | cellular process | Biological Process | 18038 |
| Total annotated genes | |  | 32194 |

Table S8. KOG annotation classification frequencies.

| KOG term | Gene number |
| --- | --- |
| RNA processing and modification | 769 |
| Chromatin structure and dynamics | 316 |
| Energy production and conversion | 909 |
| Cell cycle control, cell division, chromosome partitioning | 372 |
| Amino acid transport and metabolism | 775 |
| Nucleotide transport and metabolism | 179 |
| Carbohydrate transport and metabolism | 939 |
| Coenzyme transport and metabolism | 201 |
| Lipid transport and metabolism | 768 |
| Translation, ribosomal structure and biogenesis | 1329 |
| Transcription | 888 |
| Replication, recombination and repair | 347 |
| Cell wall/membrane/envelope biogenesis | 213 |
| Cell motility | 60 |
| Posttranslational modification, protein turnover, chaperones | 2254 |
| Inorganic ion transport and metabolism | 477 |
| Secondary metabolites biosynthesis, transport and catabolism | 838 |
| General function prediction only | 2796 |
| Function unknown | 583 |
| Signal transduction mechanisms | 1405 |
| Intracellular trafficking, secretion, and vesicular transport | 1085 |
| Defense mechanisms | 123 |
| Extracellular structures | 46 |
| Unamed protein | 1 |
| Nuclear structure | 91 |
| Cytoskeleton | 508 |
| Total annotated genes | 16307 |

Table S9. KO annotation classification frequencies.

| Pathway hierarchy1 | Pathway hierarchy2 | Gene number |
| --- | --- | --- |
| Cellular Processes | Cell growth and death | 526 |
| Cellular Processes | Cell motility | 191 |
| Cellular Processes | Cellular commiunity | 272 |
| Cellular Processes | Transport and catabolism | 1007 |
| Environmental Information Processing | Membrane transport | 117 |
| Environmental Information Processing | Signal transduction | 1678 |
| Environmental Information Processing | Signaling molecules and interaction | 23 |
| Genetic Information Processing | Folding, sorting and degradation | 1289 |
| Genetic Information Processing | Replication and repair | 253 |
| Genetic Information Processing | Transcription | 534 |
| Genetic Information Processing | Translation | 1537 |
| Metabolism | Amino acid metabolism | 1134 |
| Metabolism | Biosynthesis of other secondary metabolites | 482 |
| Metabolism | Carbohydrate metabolism | 1713 |
| Metabolism | Energy metabolism | 1167 |
| Metabolism | Glycan biosynthesis and metabolism | 259 |
| Metabolism | Lipid metabolism | 846 |
| Metabolism | Metabolism of cofactors and vitamins | 457 |
| Metabolism | Metabolism of other amino acids | 443 |
| Metabolism | Metabolism of terpenoids and polyketides | 403 |
| Metabolism | Nucleotide metabolism | 363 |
| Metabolism | Overview | 1327 |
| Metabolism | Xenobiotics biodegradation and metabolism | 174 |
| Organismal Systems | Circulatory system | 192 |
| Organismal Systems | Development | 105 |
| Organismal Systems | Digestive system | 290 |
| Organismal Systems | Endocrine system | 695 |
| Organismal Systems | Environmental adaptation | 599 |
| Organismal Systems | Excretory system | 242 |
| Organismal Systems | Immune system | 517 |
| Organismal Systems | Nervous system | 567 |
| Organismal Systems | Sensory system | 72 |
| Total annotated genes |  | 16321 |

Table S10. Up- and down-regulated DEGs in the transcriptomic comparisons of D vs. S. Padj (the adjusted *p* value) < 0.05.

In the individual excel format “Supplementary Table S10”.

Table S11. FPKM (fragments per kilobase of transcript per million base pairs sequenced) of the dwarf and standard samples.

In the individual excel format “Supplementary Table S11”.

Table S12. Differentially expressed genes related to cell division, cell elongation and phytohormone biosynthesis, metabolism and signal transduction.

In the individual excel format “Supplementary Table S12”.

Table S13. Forward and reverse primer sequences of genes evaluated using qPCR experiments.

| Gene ID | Forward primers | Reverse primers |
| --- | --- | --- |
| AUX/IAA c45786_g3 | GATGCGGTCCTGAACAAATTC | CCTTGAGGTCTACCTTTCTTCC |
| ARF c51630_g2 | GGGATGAACATGCATCAATTACC | AGATTCTGCTGATTGGGTTAGG |
| PIN c33756_g1 | AGGGACATTACTTGGAGAGGAG | GGCTTTCGGGTTACCGATTT |
| PIN c33103_g1 | CTCTTACACGTCGCCATTGT | GAGCGATCAACATCCCGAATA |
| YUCCA c44689_g1 | CTCAGGGATGGAAGTCTGTTTG | GACGTGAACCGTGTCTTTGA |
| SAUR c31498_g1 | CCGAACTTCCACTTCCATCTT | GATGTCTTTGGGCTGCTTCT |
| ILR1 c43479_g1 | TCACATCCTTCACCCATCAATC | CTGCCGGGTCTTTATGTTTCT |
| CYCD c43140_g1 | CGATCACTCCGTTCTCATTC | AGCCCAGGAGGTTAATATCT |
| EXPA c39031_g1 | CACCATCAACGGAAGAGACTAC | TTCCTGGACATTGCCATCC |
| PIN c47200_g1 | CACACAGACACACTTCCTCTT | CGCACATGACGTTGTAGAAATC |
| CYCD c47057_g1 | ACAAGACACCAAGAGAGAGATTG | CGGGTCACTGTTACTGATGAAT |
| CYCD c37709_g1 | CGAGGCCAAGACGATAAAG | GTGCGATGTGATCGAGAAA |
| CKX c44600_g2 | CAACGGTCCCATTCTCATCTAC | GGCATAGCAGACGACAGAAA |
| PP2C c48755_g2 | CCTCGAGTACGTAGCTTGTTTC | GCTGTTGTTCTCCAAACTGAAC |
| ACO c43548_g1 | ATTCTTCCAGGTGGTGAATC | GCGTACTTCTGTTTCTCATCTA |
| BKI1 c45298_g2 | AACAGGACACCATCAGAACAA | GGAGAATTCATGGGAAGGAGAG |
| GID1 c37068_g1 | CGCCGTCTAGTCAGCATTT | GCAGCCCAACCATCTTCATA |
| CKX c50290_g1 | CGGCCTGGACTACAAGATATAC | CTTCCTCTCGACGAACTTAGAC |
| LOG c38343_g1 | CATTGAACTCGGCAAGGAATTG | CGTCATAGACAGCCTGAGAGA |
| ABF c37750_g1 | AGGGATTGGGAAGGACTTTG | CACCACTAGAAGATGCCAGATAG |
| BAK1 c48408_g1 | CATTACTGCCGAGGATGTAGAA | CCTATGAGACCGAGCAGATTG |
| GH3 c43299_g1 | GTCCCGGTCATCACTTACAA | CCCGAGCTGCATAACATTTC |
| EF-1α | GACTGTGCTGTGCTCATC | GTGGCATCCATCTTGTTG |
